# Flower-like Palladium Nanoclusters Decorated Graphene Electrodes for Ultrasensitive and Flexible Hydrogen Gas Sensing

**DOI:** 10.1038/srep12294

**Published:** 2015-07-22

**Authors:** Dong Hoon Shin, Jun Seop Lee, Jaemoon Jun, Ji Hyun An, Sung Gun Kim, Kyung Hee Cho, Jyongsik Jang

**Affiliations:** 1 World Class University program of Chemical Convergence for Energy & Environment, School of Chemical and Biological Engineering, Seoul National University, 151-742, Korea

## Abstract

Flower-like palladium nanoclusters (FPNCs) are electrodeposited onto graphene electrode that are prepared by chemical vapor deposition (CVD). The CVD graphene layer is transferred onto a poly(ethylene naphthalate) (PEN) film to provide a mechanical stability and flexibility. The surface of the CVD graphene is functionalized with diaminonaphthalene (DAN) to form flower shapes. Palladium nanoparticles act as templates to mediate the formation of FPNCs, which increase in size with reaction time. The population of FPNCs can be controlled by adjusting the DAN concentration as functionalization solution. These FPNCs_CG electrodes are sensitive to hydrogen gas at room temperature. The sensitivity and response time as a function of the FPNCs population are investigated, resulted in improved performance with increasing population. Furthermore, the minimum detectable level (MDL) of hydrogen is 0.1 ppm, which is at least 2 orders of magnitude lower than that of chemical sensors based on other Pd-based hybrid materials.

Hydrogen (H_2_) gas is used extensively in many industrial processes and is an essential fuel source in clean-energy transportations and power generation applications^1^,^2^. However, it is highly flammable and explosive at volume concentrations higher than *ca.* 4%. Therefore, hydrogen sensors that have high sensitivity, rapid response, and reversibility are required to detect and/or monitor minute hydrogen leakages in industrial applications^3^,^4^. In general, commercial hydrogen sensors composed of metal oxide (SnO_2_) films meet these demand but require an operating temperature of over 200 °C, which increases the overall power consumption of the sensing device^5^,^6^,^7^.

Palladium (Pd) is an attractive candidate to replace metal oxides, because H_2_ molecules are selectively adsorbed onto the surface of Pd by dissociation into hydrogen atoms (H_2_→2H), and diffused into the interstitial sites of Pd structure. As a result, the phase of Pd transfer a solid solution of Pd/H (α-phase) and a palladium hydride (β-phase), resulted in resistance changes at room temperature. However, materials based on Pd is susceptible to structural changes (such as vacancy and dislocation), which are increased during the phase transition of Pd (α to β) that occurs at hydrogen concentration higher than 2%, causing have been known to collapse during the sensing reaction due to an irreversible phase change^8^,^9^,^10^,^11^,^12^,^13^,^14^.

The shape control of metal nanostructure is important factor to enhance the activity and stability^15^. Numerous research have studied to improve the performance by change the nano-sized morphology such as nanocube^16^, nanorod^17^,^18^, nanowire^19^, polyhedron^20^, nanoplate^21^, hollow structure^22^. Furthermore, the substrate for the introduction of these shape is also play a critical roles due to the improvement of the charge transport and stability of active materials.

Graphene, a two-dimensional materials with honeycomb structure composed of single-layer sheet of sp^2^-hybridized carbon atoms, has attracted as a substrate materials. Among these graphene substrate, CVD graphene is one of promising substrate owing to its unique physical properties (high electronic conductivity, good thermal stability), and excellent mechanical strength^23^,^24^,^25^. For example, M. G. Chung *et al.* fabricated flexible hydrogen gas sensors using CVD graphene decorated with Pd nanoparticles *via* electrodeposition^26^. W.Wu *et al.* synthesized CVD graphene on SiO_2_/Si decorated with thin Pd film using electron beam evaporation^27^. However, these approaches have limitations that are controlling the shape and population because the degree of functional groups on the graphene surface cannot be controlled.

Herein, this study demonstrates a simple strategy for fabricating flower-like palladium nanoclusters on CVD graphene (FPNCs_CG) electrode through the electrodepostion. The flower shapes are formed by modifying the graphene surface with 1,5-diaminonaphthalene (DAN). The population of FPNCs is well-controlled by adjusting the DAN concentration in the functionalization solution. Furthermore, flexibility of electrode can be obtained through transfer of CVD graphene onto PEN film. The resulting FPNCs_CG electrode films are used as signal transducer for the detection of hydrogen gas. The response of these electrode toward H_2_ gas is both sensitive and reversible, and is attributed to the more active site from flower-like shape of the Pd nanostructures and the high carrier mobility of the underlying graphene. The minimum detectable level (MDL) of H_2_ is as low as 0.1 ppm, which is considerably lower than that of other chemical sensors based on carbon-based palladium composites. To our knowledge, no previous reports have described the electrodeposition of flower-like metal nanoclusters on CVD graphene.

## Results and Discussion

### Fabrication of FPNCs_CG electrode

**Figure 1:**
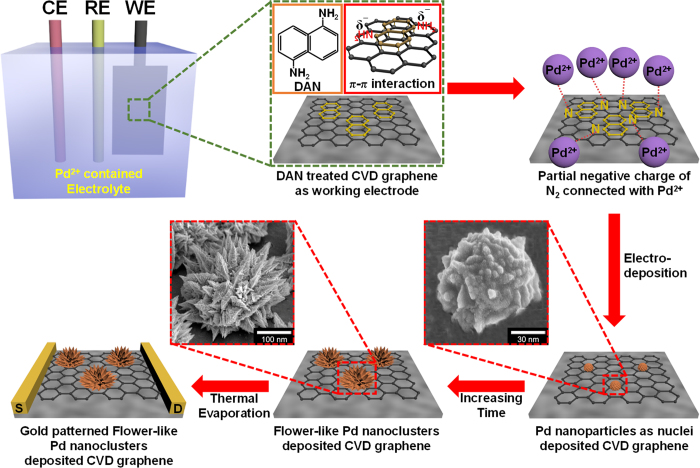
(**a**) Illustrative diagram for the fabrication steps of FPNCs_CG electrode.

**Figure 2:**
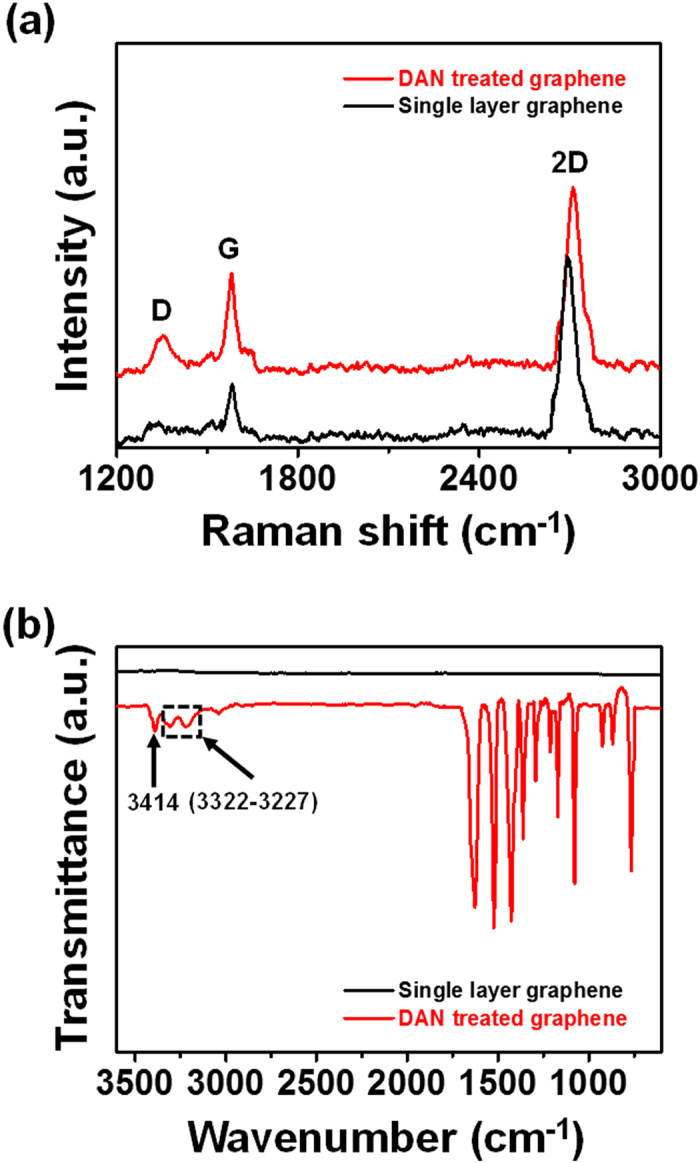
(**a**) RAMAN and (**b**) FT-IR spectrum of single layer graphene untreated (black) and treated (red) with DAN.

**Figure 3:**
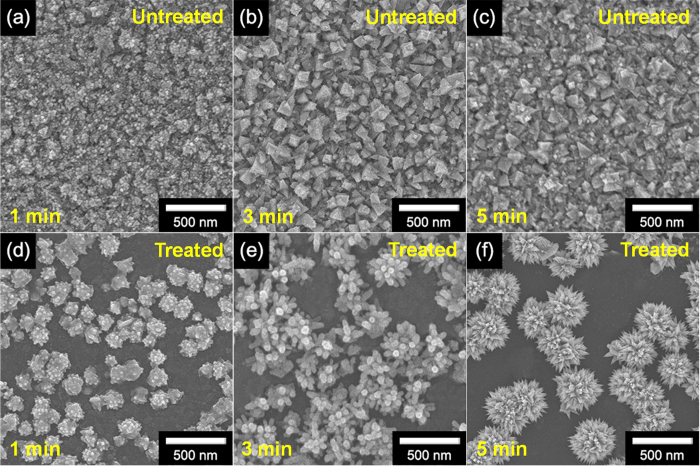
(**a**) FE-SEM images of FPNCs_CG untreated and treated with DAN for (**a**), (**d**) 1 min, (**b**), (**e**) 3 min, and (**c**), (**f**) 5 min, respectively.

Figure 1 illustrates the overall procedure for the fabrication of flower-like palladium (FPNCs) decorated CVD graphene (CG) electrodes. CG is grown on copper (Cu) foil using methane (CH_4_) as carbon source and hydrogen (H_2_) as a catalyst^28^,^29^. The Cu foil is removed with a Cu-etchant and the CG is transferred to a poly(ethylene naphthalene) (PEN) substrate for flexibility. Before the electrodeposition, the surface of the CG is chemically functionalized with amino groups by reacting with 1,5-diaminonaphthalene (DAN), which orients into flat stacks on the graphene surface due to π-π interactions between the phenyl group of DAN and aromatic structure of the graphene. To confirm the chemical functionalization of the CG surface, RAMAN spectra of DAN-treated and -untreated CG surface are shown in Fig. 2a. The D peak (1354 cm^−1^) generates after the DAN treatment, owing to the disorder of the graphene basal plane. Furthermore, The *I*_2D_/*I*_G_ intensity ratio of surfaces that is untreated with DAN (*ca.* 3.7) is higher than that of DAN-treated surfaces (*ca.* 1.8). This change suggested that charge impurity or rippling on the surface cause an inhomogeneous charge distribution and electron-donating containing aromatic molecules induced a ratio decrease due to the doping effect. Additionally, the upshift of 2D and downshift of G band wavenumber can be observed by electrical gating, owing to the dynamic effect of carrier population^30^,^31^. Furthermore, Fourier Transform InfraRed (FTIR) spectra (Fig. 2b) of CG displays the linear line, due to the atomic scale^32^,^33^. The presence of DAN on the treated graphene surface is confirmed by peaks at 1362–1293 cm^−1^, corresponding to C-N stretching vibrations of primary amino groups, and at 3414 and 3322–3227 cm^−1^, corresponding to asymmetric and symmetric N-H stretching of aromatic primary amino groups, respectively^34^,^35^. According to the FT-IR of FPNCs_CG, the DAN does not remain after electrodeposition (Fig. S3). Then, Pd is directly deposited onto the DAN-treated and -untreated graphene surface as working electrode with a Pd precursor contained sulfuric acid electrolyte in a three-electrode system. In case of DAN-untreated graphene, Pd^2+^ ions react with numerous functional group on the graphene surface induced by moisture, oxygen, and fabrication process, resulted in evenly spread hexagonal pyramid structure (Fig. 3a–c)^36^. Conversely, partial negative charges on the nitrogen atoms of DAN preferentially combine with Pd^2+^ ions than the other functional groups. Pd nanoparticles (NPs) are then deposited on the graphene surface. With increasing electrodeposition time, flower-like palladium nanoclusters (FPNCs) are formed from these Pd NPs as nucleation sites (Fig. 3d–f). FPNCs formation mechanism can be explained as following reason^37^,^38^. Sulfate ions (SO_4_^2−^) in sulfuric acid theoretically prohibit the growth of Pd at the adsorption sites. Furthermore, this effect is maximized on the Pd (111) plane, which is preferred adsorption plane for SO_4_^2−^ ions. To confirm the influence of sulfate ions, sulfuric acid as electrolyte is prepared with various concentration (0.01 and 0.1 M) as shown in Fig. S1. Smooth structure is observed at high concentration of H_2_SO_4_, due to the uniformly hindrance of the growth of (111) plane on the Pd surface. Adversely, low concentration induce to the partial hindrance, resulted in more sharp structure. This result indicate that Pd is predominantly composed of (111) plane and the growth of Pd can be controlled by sulfate ions concentration.

**Figure 4:**
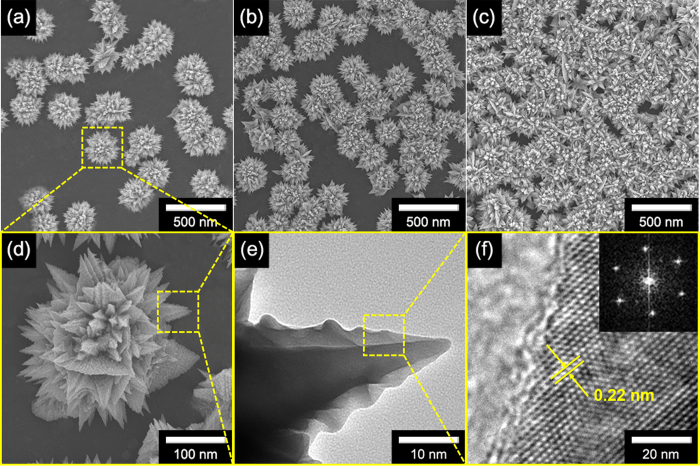
FE-SEM images of FPNCs_CG with various populations of FPNCs ((**a**) low, (**b**) medium, and (**c**) high). (**d**) High-resolution SEM, (**e**) TEM images, (**f**) HR-TEM and FFT pattern (inset) of FPNCs_CG.

FE-SEM micrographs of FPNC_CG surfaces with various populations of FPNCs are shown in Fig. 4a–c. The population of FPNCs increase with increasing DAN concentration from 0.01 to 0.1 M, suggesting that the degree of modification is proportional to the number of Pd NPs as nuclei. In addition, the FPNCs are approximately *ca.* 300 nm in size and compose of numerous needles of hexagonal pyramids (Fig. 4d) with *ca.* 80 nm in length (Fig. 4e). The HR-TEM images in Fig. 4f shows that the FPNCs are highly crystallized, as indicated by well-defined fringe patterns. The corresponding FFT diffractogram shows a six-fold symmetry of diffraction spots indicative of hexagonal faces bound by the (111) plane, as described above. In addition, the interplanar spacing calculated from the FFT analysis of the micrograph is *ca.* 0.22 nm, which is consistent with the atomic spacing of (111) face-centered-cubic (fcc) Pd^16^,^17^,^18^,^19^,^20^,^39^. The FPNCs deposited on CG with functionalization solutions containing 0.01, 0.1, and 1 M DAN are denoted FPNCs_CG_L, FPNCs_CG_M, and FPNCs_CG_H, respectively.

### Character of FPNCs_CG electode

**Figure 5:**
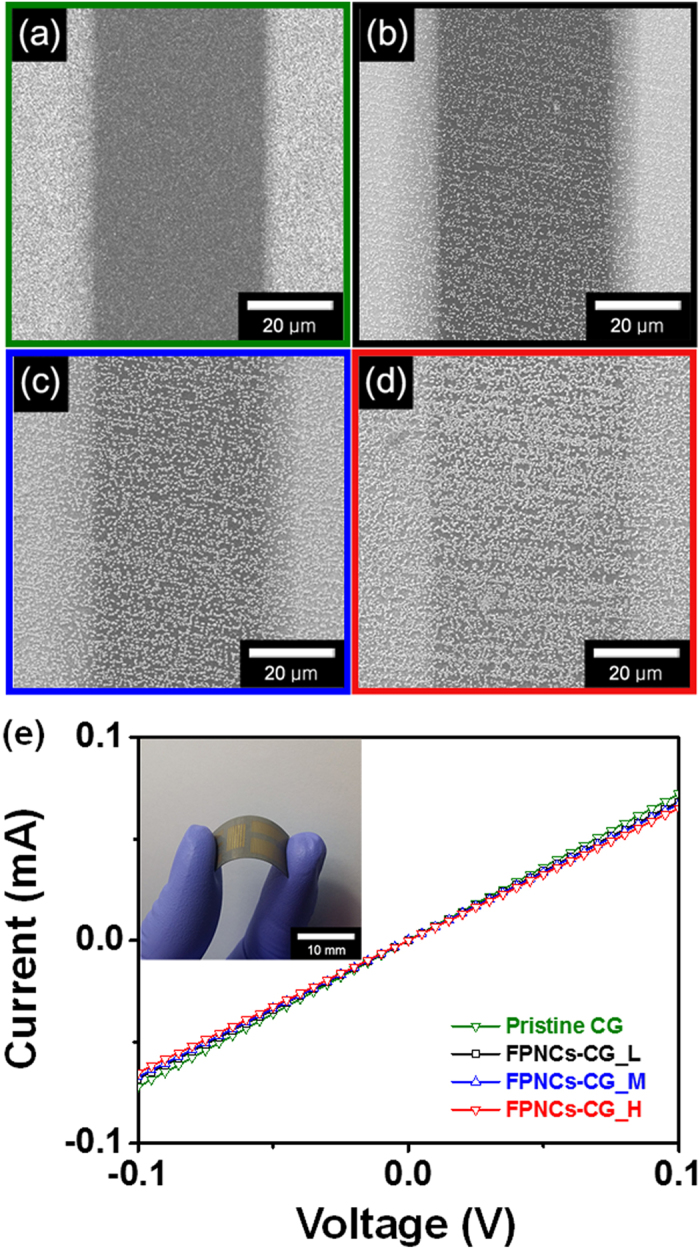
FE-SEM images of (**a**) Pristine CG, (**b**) FPNCs_CG_L, (**c**) FPNCs_CG_M, and (**d**) FPNCs_CG_H deposited on gold electrode, respectively. (**e**) Real images of flexible electrode deposited with the gold pattern (inset) and *I-V* curves of Pristine CG (green), FPNCs_CG_L (black), FPNCs_CG_M (blue), and FPNCs_CG_H (red).

Figure 5a–d show FE-SEM micrographs of gold deposited FPNCs_CG electrode with various populations of FPNCs, demonstrating that the population of FPNCs can be well controlled. Figure 5e shows current-voltage (*I–V*) curves of the FPNCs_CG films and indicates that the FPNCs are in ohmic contact with graphene surface. Contact resistance increases slightly with increasing of the FPNCs population, implying that the FPNCs acts as impurity for decrease of quality. However, this slight change in contact resistance may be ignored in practical applications due to the overall high conductivity of the FPNCs_CG films.

Powder X-Ray Diffraction (XRD) of the FPNCs_CG films contains an intense and sharp peak corresponding to graphitic carbon (002) at about 2*θ *= 24°, suggesting that the single layer of CVD graphene is highly crystalline (Fig. S2a). XRD spectrum of the FPNCs are consistent with those of standard materials (JCPDS 40835). The formation of FPNCs is confirmed by diffraction peaks at *2θ *= 40.12, 46.3, 67.8 and 81.6, corresponding to (111), (200), (220), and (311) reflections of the *fcc* lattice. Energy Dispersive X-ray (EDX) spectra revealed that the FPNCs are composed of Pd and carbon elements, implying that the FPNCs are deposited on the graphene surface (Fig. S2b). The chemical composition of FPNCs_CG films is investigated by X-ray Photoelectron Spectroscopy (XPS). Figure S2c displays the full spectra acquired from 0 to 1000 eV. Only C, O, and Pd are present. The Pd 3d peak can be accurately fit with two prominent peaks at 335 and 341 eV, indicating that the Pd^2+^ ions are completely reduced to Pd^0^ during the electrodeposition process (Fig. S2d).

### Real-time responses of Hydrogen gas sensor

**Figure 6:**
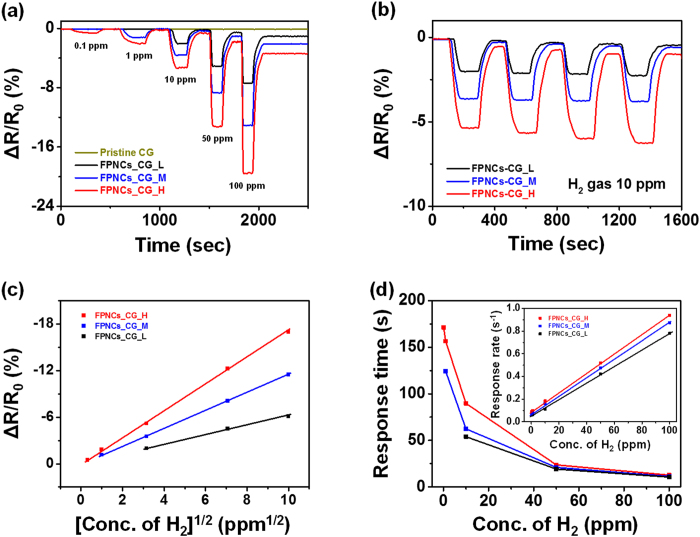
Reversible and reproducible responses are measured with various populations of FPNCs_CG at a constant current value (10^−4^ A). Nomalized resistance changes at room temperature upon (**a**) sequential exposure to H_2_ gas of various concentrations (0.1 to 100 ppm) and (**b**) periodic exposure to H_2_ gas (10 ppm) of Pristine CG (green), FPNCs_CG_L (black), FPNCs_CG_M (blue), and FPNCs_CG_H (red), respectively. (**c**) Sensitivity as a function of square root of H_2_ concentration, (**d**) response time and rate (inset) as a variation of H_2_ concentration.

**Figure 7:**
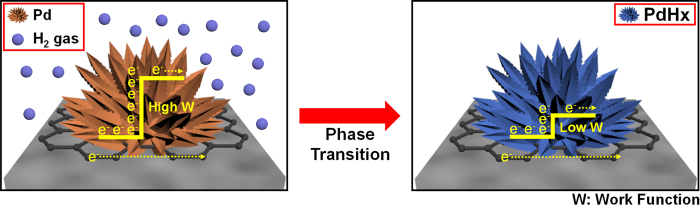
Scheme of hydrogen gas sensing mechanism of FPNCs_CG.

Figure 6 shows the real-time response of a FPNCs_CG electrode to an H_2_ atmosphere at room temperature. The resistance change, *ΔR*, of the electrode is monitored during sequential or periodic exposures to H_2_. *ΔR/R*_*0*_ (sensitivity) is defined as the percent resistance change upon exposure to a gas with a fixed concentration of hydrogen and is calculated as follows:


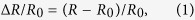


where *R*_*0*_ is the resistance of the sensor exposed to dry air and *R* is the maximum resistance after exposure to a gas containing hydrogen. When the FPNCs_CG is exposed to hydrogen gas, the phase of the FPNCs is transfered from palladium (Pd) to palladium hydride (PdHx). Consequently, decreasing of the work function is beneficial to the flow of more electrons, resulted in decreasing of the resistance (Fig. 7)^40^,^41^,^42^. Resistance of the films is measured in real time upon exposure to various concentrations of H_2_ gas. In contrast to pristine CG, all of the FPNCs_CG sensors exhibit a rapid and reversible response to H_2_ gas at room temperature. Figure 6a shows the electode response during sequential exposures of increasing analyte concentration (0.1, 1, 10, 50, and 100 ppm). Sensitivity increases with increasing populations of FPNCs, indicating that the population of FPNCs is active materials to react with hydrogen atoms. The minimum detectable level (MDL) of H_2_ measured with the FPNCs_CG_H (0.1 ppm) is lower than that of FPNCs_CG_L (10 ppm). This is likely because higher populations of FPNCs provide more reactive sites for hydrogen atoms. Furthermore, the detection limit of FPNCs_CG_H is three orders of magnitude lower than that of noble metal/graphene based electrodes (Table S1). The electrical response of a FPNCs_CG electrode upon periodic exposures to 10 ppm H_2_ is shown in Fig. 6b. Testing over five cycle yields similar responses and sensitivity without abatement. Furthermore, the sensitivity is directly proportional to the square root of H_2_ concentration as shown in Fig. 6c. This can be explained by the Langmuir adsorption isotherm theory about dissociates of hydrogen molecule upon adsorption on the Pd surface^18^,^43^. The H_2_ dissociation reaction on Pd surface can be describes as


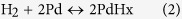


The adsorption and desorption rate of hydrogen are 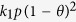 and 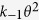 (where, 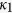 and 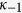 are the adsorption and desorption constant, respectively). 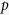 is partial pressure of H_2_, and 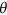 is the fraction of Pd covered by hydrogen that is proportionate to sensitivity 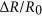. The adsorption rate equals the desorption rate at equilibrium,


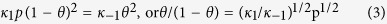


At low concentration of H_2_ 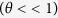, the sensitivity is proportional to the square root of the partial pressure and concentration of H_2_ as follows.


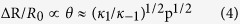


The response time (τ) as a function of hydrogen concentration is shown in Fig. 6d. The response time is increased with increasing population of FPNCs, indicated that large active sites are required more time to saturate. Furthermore, reciprocal of the response time 1/τ follows a linear correlation with hydrogen concentration. The adsorption rate of H_2_ is 
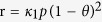
, and *θ* is negligible at the low concentration. Therefore, the adsorption rate is estimated to be 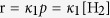, which is well-matched in inset of Fig. 7d.

The electrodes were operated in dry air (using only N_2_ flow; humidity: at below ca. 10%), however, the humidity was important factor for sensitivity and response/recovery time. The sensing performance was measured at wet air (using bubbled water with N_2_ flow; humidity: ca. 30%) to investigate the effect of humidity. The resistance of FPNCs_CG electrodes decreased at both water drop and bubbled water, implying that water obviously influenced the resistance (Fig. S4a and b). When the exposed to hydrogen gas with wet air, the strong peak and fast response/recovery time could be confirmed compared to drying condition (Fig. S4c). This is because functional groups related to oxygen on the graphene surface is preferentially adsorbed with water vapor and decreases the p-type doping, resulted in decrease of the resistance^44^,^45^,^46^.

The FPNCs_CG based sensors also exhibited high selectivity for H_2_ gas compared to nitrogen dioxide (NO_2_) and ammonia (NH_3_) gas (Fig. S5). When the pristine CG was exposed to NO_2_ gas, the resistance decreased (Fig. S5a). On the other hands, the resistance upon exposure to NH_3_ gas shows increasing tendency (Fig. S5b). This is because NO_2_ and NH_3_ gases is electron acceptors and electron donor gas, resulted in change the hole concentration of graphene (p-type)^47^,^48^,^49^,^50^. According to the deposition of FPNCs, the resistance toward both gases increased, due to the reduced carrier mobility by the scattering effect of Pd. Furthermore, Sensitivity and MDL of NO_2_ and NH_3_ were lower than that of hydrogen gas, due to the different sensing mechanism of hydrogen (phase transition), indicating that FPNCs_CG based sensor show the high selectivity^51^.

**Figure 8:**
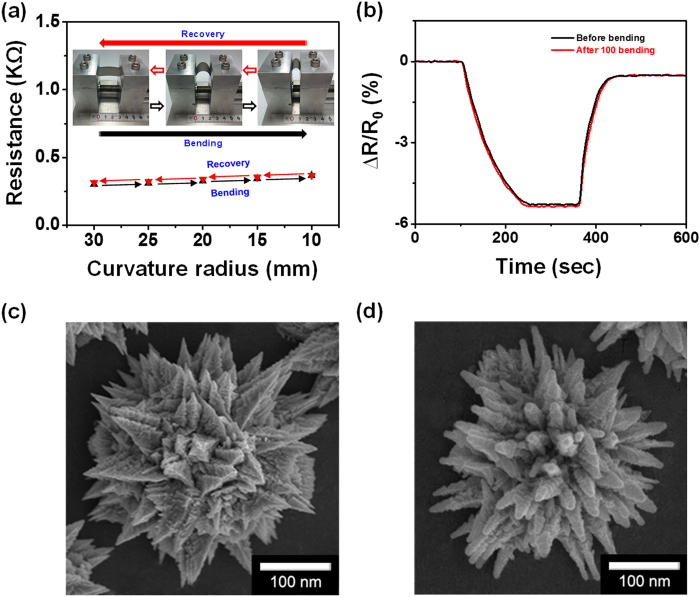
(**a**) Resistance changes of flexible H_2_ sensor electrode for different curvature radius, and (**b**) Sensing behavior of the H_2_ gas (10 ppm) before and after 100 bending of FPNCs_CG_H. FE-SEM images of FPNCs_CG (**c**) before (**d**) after H_2_ gas sensing for 100 cycles.

The resistance of the FPNCs_CG electrode depended on the bend radius of the film-substrate assembly. Therefore, the resistance of the FPNCs_CG as a function of bend radius, shown in Fig. 8a, is used to evaluate the mechanical stability of FPNCs_CG sensors on PEN substrates^43^,^52^,^53^,^54^. No significant change in resistance is observed down to a bend radius of *ca.* 10 mm. Figure 8b shows the change in sensor response after repeated bending and relaxing. The response decreases by only 2% after 100 bending cycles. Furthermore, the morphology of the FPNCs is maintained without collapse even after 100 cycles of H_2_ exposure (Fig. 8c,d). This demonstrates the excellent mechanical flexibility and durability of the FPNCs_CG sensor electodes and shows that these film may be useful in wearable sensors^55^,^56^,^57^,^58^.

## Conclusion

In conclusion, flower-like palladium nanoclusters (FPNCs) are electrodeposited on CVD graphene (FPNCs_CG) electrode. The shape and population of FPNCs can be controlled by modifying the graphene surface with DAN. The phenyl groups of DAN interact with the graphene surface v*ia* π-π interactions. The amino groups of DAN bind with Pd^2+^ ions to form Pd nanoparticles that act as nucleation sites. These sites, and the hindering effects of sulfate ions, resulted in the growth of FPNCs on the graphene surface. Transferring the CVD graphene film onto PEN substrates provide sensor films with excellent flexibility and desirable mechanical properties. The FPNCs_CG electrode are used as the signal-transducing element in hydrogen gas sensors at room temperature. The sensitivity and response time of these sensors improve with increasing FPNCs population. In particularly, the FPNCs_CG_H electrode has a minimum detectable level (MDL) of 0.1 ppm H_2._ This report describes an effective method for the fabrication of flower-like metal-graphene composites with population control for various flexible electrochemical applications.

## Methods

### Materials

1,5-diaminonaphthalene (DAN), palladium (II) chloride (PdCl_2_), and sulfuric acid (H_2_SO_4_) were purchased from Aldrich Chemical Co. and used as received.

### Fabrication of CG on the flexible film

Graphene was synthesized by chemical vapor deposition (CVD) on copper (Cu) foil in a CH_4_/H_2_ atmosphere in a furnace chamber. The Cu foil was placed in the furnace chamber and H_2_ gas was introduced at a flow rate of 8 sccm and a pressure of 147 mTorr for 30 min to stabilize the gas flow. The furnace was then heated at 40 °C/min to 1000 °C and held for 30 min. CH_4_ gas was introduced at 20 sccm and 560 mTorr and the chamber was cooled to 200 °C after 30 min. After the surface of the graphene was coated using PMMA solution, the Cu foil and associated impurities were removed using a copper etchant and 0.01 M sulfuric acid (H_2_SO_4_), respectively. Finally, the graphene was transferred to a flexible polyethylene naphthalate (PEN) film and dipped into the acetone solution at 60 °C for 2 hr to remove the PMMA.

### Synthesis of FPNCs_CG electrode

Electrolyte for the electrodeposition of FPNCs was prepared by dissolving 0.1 M PdCl_2_ into 100 mL of 0.05 M H_2_SO_4_. Prior to electrodeposition, as-prepared CVD graphene piece (1.5 cm × 1.5 cm) was immersed in a methanolic solution containing a certain concentration (0.01, 0.1 and 1 M) of DAN (1,5-diaminonaphthalene) for 30 min. The concentration of DAN was used as the variable in controlling the population of deposited FPNCs. The electrodeposition process was conducted with a three-electrode system: the DAN-treated CVD graphene was used as the working electrode with a Ag/AgCl reference electrode and a Pt foil counter electrode. Pd was deposited by applying a constant potential of −0.1 V for 10 min. Then, the gold electrode was deposited on the FPNCs_CG by thermal evaporation.

### Characterization of FPNCs_CG

Field Emission Scanning Electron Microscope (FE-SEM) images were obtained using a JEOL 6700 instrument. JEOL JEM-200CX and JEOL JEM-3010 instruments were used for Transmission Electron Microscopy (TEM) and High-Resolution Transmission Electron Microscopy (HR-TEM), respectively. Samples were dispersed in ethanol and cast onto perforated carbon grids. X-ray Photoemission Spectroscopy (XPS) and X-Ray Diffraction (XRD) experiments performed on a JPS-9000MS (JEOL, Mg Kα X-ray source) and M18XHF-SRA (Rigaku, SmartLab, λ = 1.5418 Å) instruments, respectively. RAMAN spectra were recorded on an FRA 1106/S FT-Raman (Bruker) spectrometer and excited with a 514-nm Ar laser. Samples for RAMAN spectroscopy were prepared on silicon-oxide-treated silicon wafers. The electrical properties and sensing performance of the FPNC_CG films were investigated using a current-source meter (Keithley 2400). I–V characteristics were determined using a WBCS 3000 potentiostat (WonA Tech). Fourier-Transform InfraRed (FT-IR) spectra were acquired using a Frontier FT-IR spectrometer (Perkin Elmer Inc.).

### Electrical measurements of FPNCs_CG sensor

Resistance changes in the FPNC_CG films were monitored with a source meter connected to a computer. The FPNC_CG sensors were placed in a vacuum chamber within a vapor inlet/outlet pressure of 100 Torr. Hydrogen (H_2_) gas at various concentrations (0.1–100 ppm) was introduced into the chamber using a mass flow controller (MFC, KNH Instruments). The real-time resistance was measured at a constant applied current of 10^−4^ A. After exposure, the H_2_ gas was removed by blowing across the FPNC_CG film with compressed air (N_2_). This process was repeated several times. H_2_ gas/N_2_ air mixtures were supplied at various flow rates of 1–5 sccm and 2–8 slm using a MFC controller.

## Additional Information

**How to cite this article**: Shin, D. H. *et al.* Flower-like Palladium Nanoclusters Decorated Graphene Electrodes for Ultrasensitive and Flexible Hydrogen Gas Sensing. *Sci. Rep.* **5**, 12294; doi: 10.1038/srep12294 (2015).

## Supplementary Material

Supplementary Information
