# Supplementary material for: Flower-like Palladium Nanoclusters Decorated Graphene Electrodes for Ultrasensitive and Flexible Hydrogen Gas Sensing
Source: Sci Rep. 2015 Jul 22;5:12294. doi: 10.1038/srep12294 (PMC4648453; doi:10.1038/srep12294)
Supplement: Supplementary Information [file srep12294-s1.pdf]

Supplementary Information for:

Flower-like Palladium Nanoclusters Decorated Graphene Electrodes for Ultrasensitive and Flexible Hydrogen Gas Sensing

Dong Hoon Shin, Jun Seop Lee, Jaemoon Jun, Ji Hyun An, Sung Gun Kim, Kyung Hee Cho and Jyongsik Jang[§]

World Class University program of Chemical Convergence for Energy & Environment, School of Chemical and Biological Engineering, Seoul National University, 151-742, Korea

[§]Correspondence and requests for materials should be addressed to J.J. (jsjang@plaza.snu.ac.kr).

[*] E-mail: jsjang@plaza.snu.ac.kr
Tel.: +82-2-880-7069
Fax: +82-2-888-1604

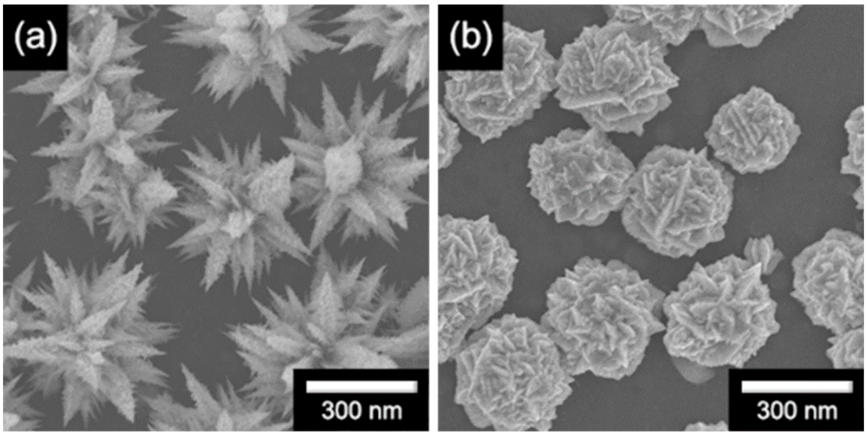

Figure S1. FE-SEM images of FPNCs_CG using sulfuric acid of (a) 0.01 M and (b) 0.1 M as electrolyte.

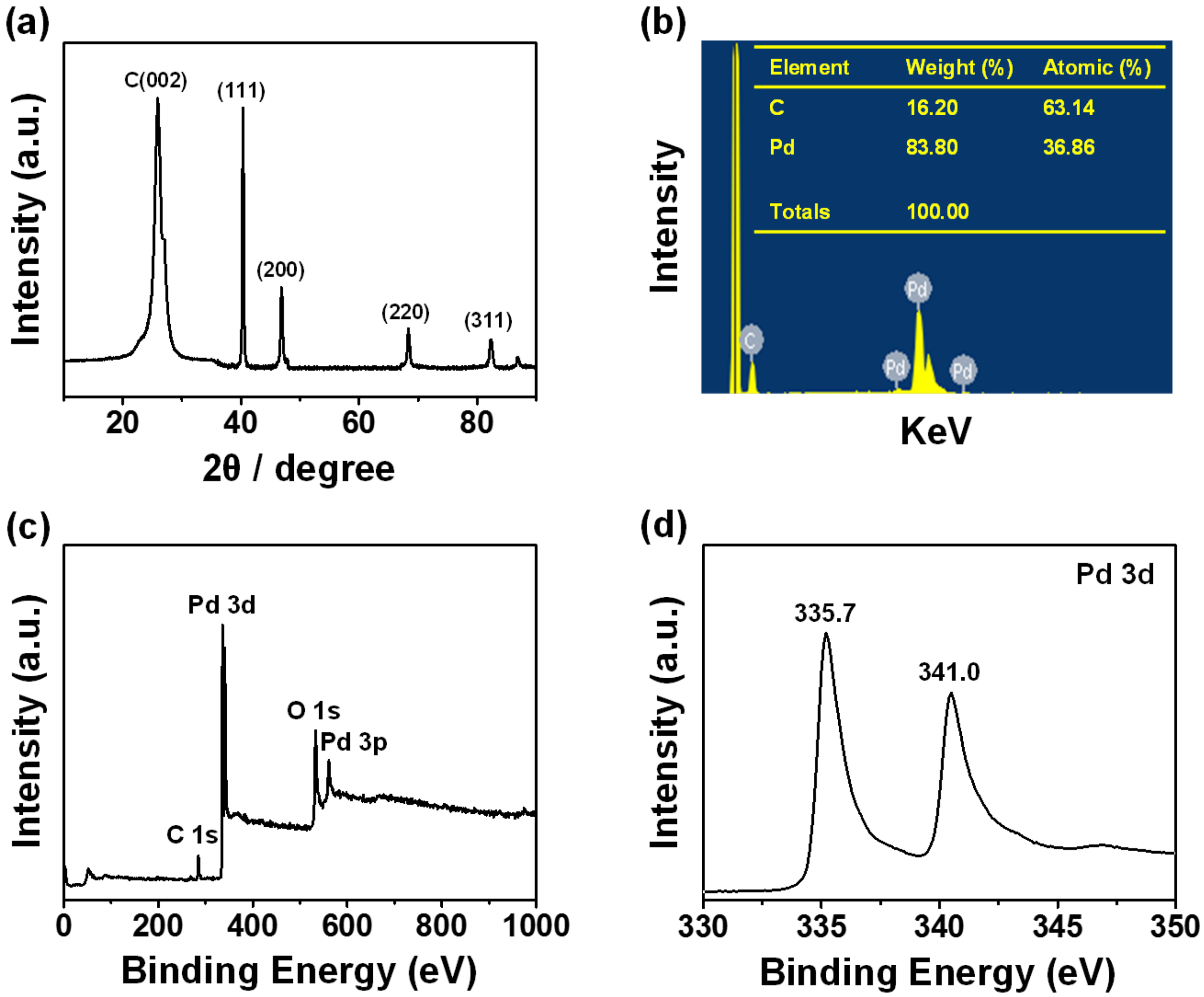

Figure S2. (a) XRD patterns, (b) EDX spectrum, and XPS spectra: (c) fully spectrum and (d) Pd spectrum of FPNCs_CG.

Table R1. Hydrogen sensing performance of this work compared with the literature.

Sensing materials	MDL[a]	Reference
Pd NPs/Graphene nanoribbon	40 ppm	54
Pd/rGO	1 %	26
Pd NPs/CVD graphene	1000 ppm	55
Pd-doped reduced graphene oxide	200 ppm	56
FPNCs_CG	0.1 ppm	This work

a Minimum Detectable Level.

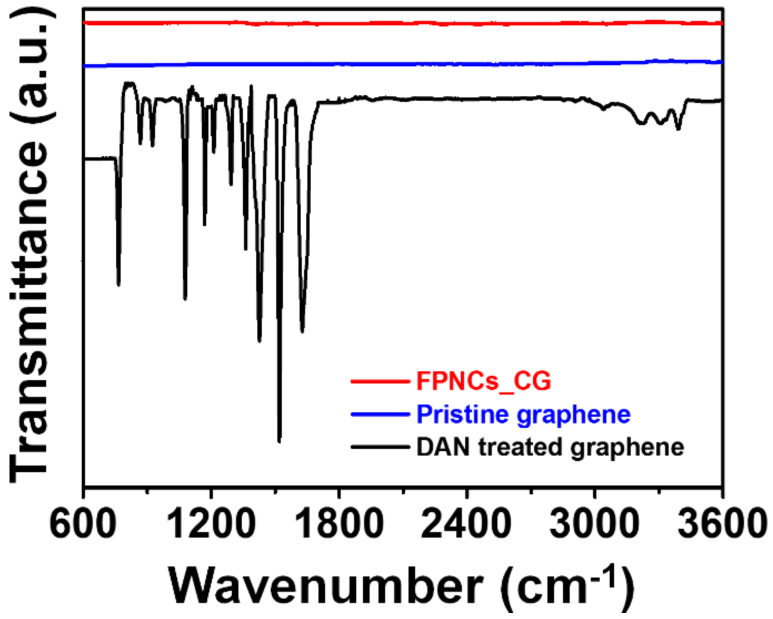

Figure S3. FT-IR spectra of DAN treated graphene (black), Pristine graphene (blue) and FPNCs_CG (red).

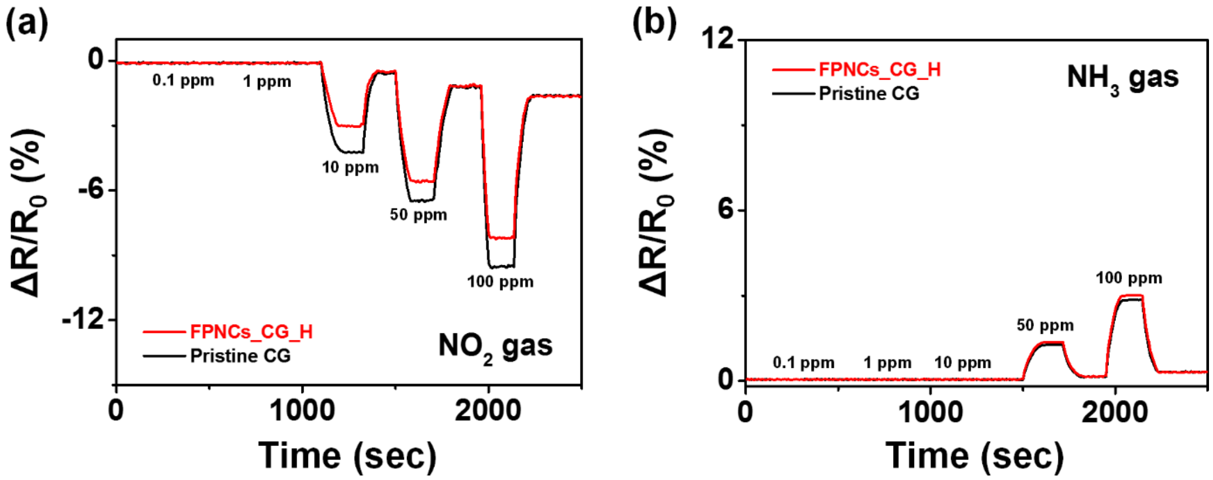

Figure S4. Reversible and reproducible responses are measured at a constant current value (10^{-4} A). Nomalized resistance changes at room temperature upon sequential exposure to (a) NO_2 and (b) NH_3 gas of various concentration (0.1 to 100 ppm) with Pristine CG (green) and FPNCs_CG_H (red), respectively.

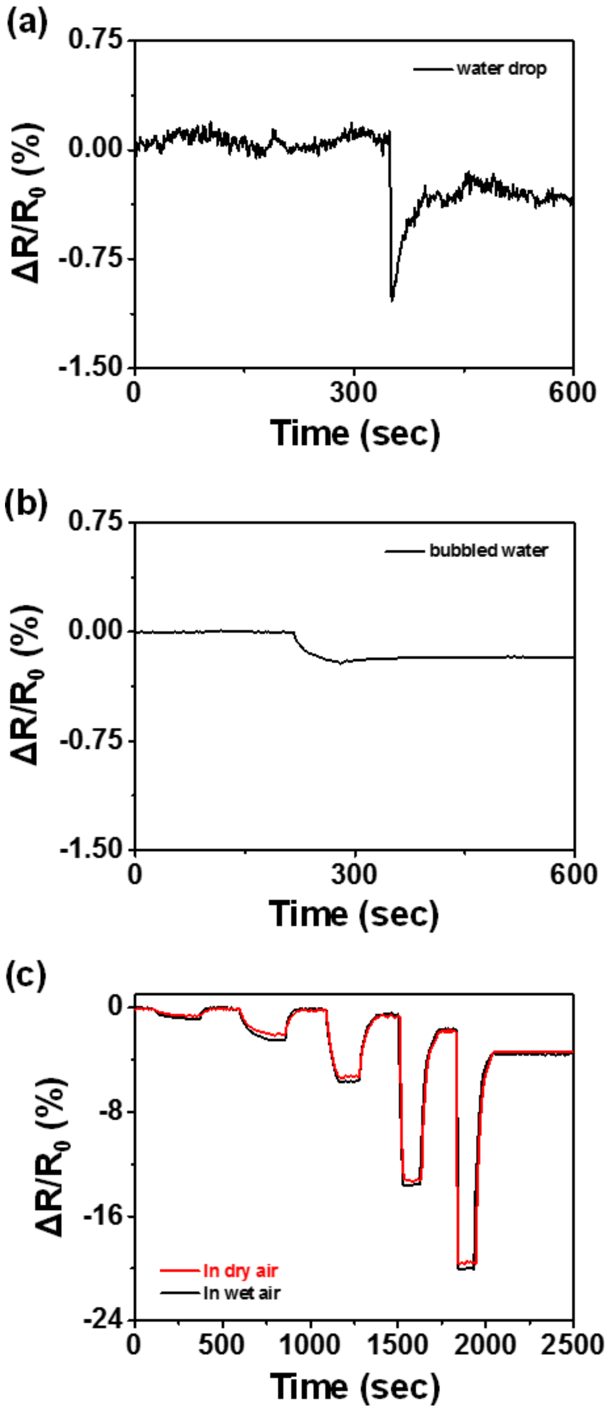

Figure S5. Reversible and reproducible responses are measured with FPNCs_CG_H at a constant current value (10^{-4} A). Nomalized resistance changes at room temperature upon exposure to H_2 gas with (a) bubbled water, (b) water drop, and (c) sequential exposure to H_2 gas of various concentration (0.1 to 100 ppm) in dry (red) and wet (black) air.
